# Supplementary material for: Stable long-term individual differences in 50-kHz vocalization rate and call subtype prevalence in adult male rats: Comparisons with sucrose preference
Source: PLoS One. 2022 Oct 27;17(10):e0276743. doi: 10.1371/journal.pone.0276743 (PMC9612506; doi:10.1371/journal.pone.0276743)
Supplement: S1 Table — SP and USVs were both measured in Phase 1 only. The relative (i.e., percentage, not absolute) prevalence of the 50-kHz call subtypes were used in the analysis. Spearman rho critical values for n = 24 are: 0.409 for p<0.05, and 0.537 for p<0.01 (2-tailed). The alpha level was set at p < 0.01 (2-tailed). (DOCX) [file pone.0276743.s001.docx]

# Supporting Information

# S1 Table Spearman rho (*r_s_*) correlation coefficients relating sucrose preference to the relative prevalence of 50-kHz call subtypes in Phase 1

| **Subtype** | **Spearman rho (*r_s_*)** |
| --- | --- |
| Complex | 0.19 |
| Upward ramp | -0.15 |
| Downward ramp | 0.20 |
| Flat | -0.08 |
| Short | 0.36 |
| Split | -0.19 |
| Step up | -0.09 |
| Step down | -0.21 |
| Multi-step | -0.02 |
| Trill | -0.18 |
| Flat-trill | -0.35 |
| Trill with jumps | -0.16 |
| Inverted-U | 0.39 |
| Composite | -0.01 |

Notes: SP and USV-related prevalence variables were both measured in Phase 1 only. The relative (i.e., percentage, not absolute) prevalences of the 50-kHz call subtypes were used in the analysis. Spearman rho critical values for n = 24 are: 0.409 for p<0.05, and 0.537 for p<0.01 (2-tailed). The alpha level was set at p < 0.01 (2-tailed). No observed values were statistically significant.
