# Supplementary material for: Stable long-term individual differences in 50-kHz vocalization rate and call subtype prevalence in adult male rats: Comparisons with sucrose preference
Source: PLoS One. 2022 Oct 27;17(10):e0276743. doi: 10.1371/journal.pone.0276743 (PMC9612506; doi:10.1371/journal.pone.0276743)
Supplement: S2 Table — The relative (i.e., percentage, not absolute) prevalence of the 50-kHz call subtypes were used in the analysis. The alpha level was set at p < 0.01 (2-tailed). (DOCX) [file pone.0276743.s002.docx]

# Supporting Information

# S2 Table Spearman rho (*r_s_*) correlation coefficients and Fisher combined p values for inter-call subtype relationships

| **Compare** | **Subtype name** | **Spearman Rho Phase 1 (*r_s_*)** | **Spearman Rho Phase 2 (*r_s_*)** | **p value Phase 1** | **p value Phase 2** | **Fisher combined**  **p value*** |
| --- | --- | --- | --- | --- | --- | --- |
| **Approach 1** |  |  |  |  |  |  |
| Trill *vs.* | flat | -0.7530 | -0.7330 | 0.0090 | 0.0132 | 0.0012 |
| Trill *vs.* | non-trill/non-flat | -0.8235 | -0.6496 | 0.1054 | 0.5072 | 0.2100 |
| Flat *vs.* | non-trill/non-flat | 0.4191 | 0.0817 | 0.0004 | 0.0172 | 0.0001 |
|  |  |  |  |  |  |  |
| **Approach 2** |  |  |  |  |  |  |
| Flat *vs.* | complex | 0.1991 | -0.0939 | 0.3509 | 0.6625 |  |
| Flat *vs.* | upward ramp | 0.5930 | 0.3096 | 0.0023 | 0.141 | 0.0029 |
| Flat *vs.* | downward ramp | -0.1765 | 0.4537 | 0.4093 | 0.026 |  |
| Flat *vs.* | flat | n/a | n/a | n/a | n/a |  |
| Flat *vs.* | short | 0.2496 | 0.3487 | 0.2396 | 0.0943 |  |
| Flat *vs.* | split | 0.3528 | 0.6786 | 0.0908 | 0.0003 | 0.0003 |
| Flat *vs.* | step up | 0.1870 | 0.4078 | 0.3817 | 0.0479 |  |
| Flat *vs.* | step down | 0.0287 | 0.2583 | 0.8941 | 0.223 |  |
| Flat *vs.* | multistep | 0.2409 | 0.2557 | 0.2569 | 0.2279 |  |
| Flat *vs.* | trill | n/a | n/a | n/a | n/a |  |
| Flat *vs.* | flat-trill | 0.1070 | 0.0261 | 0.6189 | 0.9037 |  |
| Flat *vs.* | trill with jumps | -0.5735 | -0.5223 | 0.0034 | 0.0088 | 0.0003 |
| Flat *vs.* | inverted-U | -0.5887 | -0.4626 | 0.0025 | 0.0228 | 0.0006 |
| Flat *vs.* | composite | -0.5116 | -0.1482 | 0.0106 | 0.4895 | 0.0325 |
| Flat *vs.* | unclear | -0.4678 | -0.5452 | 0.0212 | 0.0059 | 0.0012 |
| Flat *vs.* | miscellaneous | 0.0227 | 0.3163 | 0.9161 | 0.1321 |  |
| Trill *vs.* | complex | -0.0235 | 0.1826 | 0.9132 | 0.3931 |  |
| Trill *vs.* | upward ramp | -0.4983 | -0.2165 | 0.0132 | 0.3096 | 0.0266 |
| Trill *vs.* | downward ramp | 0.3322 | -0.3046 | 0.1128 | 0.1479 |  |
| Trill *vs.* | flat | n/a | n/a | n/a | n/a |  |
| Trill *vs.* | short | -0.2626 | -0.0696 | 0.2151 | 0.7467 |  |
| Trill *vs.* | split | -0.0690 | -0.2811 | 0.7487 | 0.1834 |  |
| Trill *vs.* | step up | -0.2157 | -0.3157 | 0.3115 | 0.133 |  |
| Trill *vs.* | step down | 0.1078 | -0.1696 | 0.616 | 0.4283 |  |
| Trill *vs.* | multistep | -0.3609 | -0.2670 | 0.0832 | 0.2073 |  |
| Trill *vs.* | trill | n/a | n/a | n/a | n/a |  |
| Trill *vs.* | flat-trill | -0.0670 | -0.0826 | 0.7559 | 0.7012 |  |
| Trill *vs.* | trill with jumps | 0.5231 | 0.2358 | 0.0087 | 0.2673 | 0.0164 |
| Trill *vs.* | inverted-U | 0.5487 | 0.4087 | 0.0055 | 0.0474 | 0.0024 |
| Trill *vs.* | composite | 0.4620 | 0.0576 | 0.023 | 0.7892 |  |
| Trill *vs.* | unclear | 0.5939 | 0.4983 | 0.0022 | 0.0132 | 0.0003 |
| Trill *vs.* | miscellaneous | -0.1301 | -0.1657 | 0.5445 | 0.4391 |  |

* Fisher combined p values are only shown for statistically significant (< 0.01) or trending (0.01<p<0.05) results.
