# Supplementary material for: Stable long-term individual differences in 50-kHz vocalization rate and call subtype prevalence in adult male rats: Comparisons with sucrose preference
Source: PLoS One. 2022 Oct 27;17(10):e0276743. doi: 10.1371/journal.pone.0276743 (PMC9612506; doi:10.1371/journal.pone.0276743)
Supplement: S3 Table — The coefficient of variation was calculated for 23 studies in the literature, for both the “stressed” and control groups. The mean, SD and N were stated in the text of the study, or could be estimated from a figure. (DOCX) [file pone.0276743.s003.docx]

# Supporting Information

# S3 Table Coefficient of variation values for sucrose preference tests

| **#** | **Reference** | **Control** | | | | **Experimental** | | | |
| --- | --- | --- | --- | --- | --- | --- | --- | --- | --- |
|  |  | **Mean** | **SD** | **N** | **CV** | **Mean** | **SD** | **N** | **CV** |
| 1 | Willner (1) | 70 | 16.6 | 11 | 0.24 | 45 | 16.6 | 11 | 0.37 |
| 2 | Rygula, Abumaria (2) | 72.5 | 7.1 | 8 | 0.10 | 55.5 | 8.5 | 8 | 0.15 |
| 3 | Bekris, Antoniou (3) | 77 | 8.9 | 20 | 0.12 | 40 | 22.4 | 20 | 0.56 |
| 4 | Forbes (4) | 79 | 17.9 | 20 | 0.23 | 65 | 22.4 | 20 | 0.34 |
| 5 | You, Luo (5) | 96 | 10.4 | 12 | 0.11 | 79 | 6.9 | 12 | 0.09 |
| 6 | Sun, Liu (6) | 80 | 13.9 | 12 | 0.17 | 42 | 34.6 | 12 | 0.8 |
| 7 | Luo, An (7) | 92 | 12.7 | 10 | 0.14 | 50 | 26.5 | 7 | 0.53 |
| 8 | Hamani, Machado (8) | 82 | 7.9 | 10 | 0.10 | 15 | 6.0 | 9 | 0.40 |
| 9 | Lu, Ho (9) | 70 | 24.5 | 6 | 0.4 | 45 | 30.0 | 9 | 0.7 |
|  | Studies 1-9 |  |  |  | Mean = 0.17 |  |  |  | Mean = 0.44 |
| 10 | Li, Yuan (10) | 80 | 6.9 | 12 | 0.09 | 50 | 13.9 | 12 | 0.23 |
| 11 | Chen, Wang (11) | 89 | 3.5 | 12 | 0.04 | 65 | 10.4 | 12 | 0.16 |
| 12 | Gill, Kinra (12) | 77 | 7.4 | 6 | 0.10 | 22.5 | 2.94 | 6 | 0.13 |
| 13 | Song (13) | 54 | 15.0 | 12 | 0.19 | 24 | 14.0 | 12 | 0.28 |
| 14 | Du, Wang (14) | 82 | 7.0 | 9 | 0.09 | 57 | 5.0 | 12 | 0.09 |
| 15 | Du, Zhao (15) | 80 | 10.0 | 12 | 0.13 | 60 | 7.0 | 12 | 0.12 |
| 16 | Hui, Xi (16) | 77 | 7.0 | 6 | 0.09 | 46 | 6.0 | 6 | 0.13 |
| 17 | Qin, Fang (17) | 93 | 2.4 | 9 | 0.03 | 63 | 7.4 | 9 | 0.12 |
| 18 | Su, Yang (18) | 84 | 17.3 | 12 | 0.21 | 63 | 13.9 | 12 | 0.22 |
| 19 | Wu, Ren (19) | 86 | 6.9 | 12 | 0.81 | 62 | 10.4 | 12 | 0.17 |
| 20 | Xi, Hui (20) | 80 | 8.0 | 6 | 0.09 | 50 | 7.0 | 6 | 0.11 |
| 21 | Xiong, Yang (21) | 87 | 2.3 | 8 | 0.03 | 69 | 3.9 | 8 | 0.06 |
| 22 | Yang, Liu (22) | 82.25 | 10.6 | 12 | 0.13 | 59.51 | 9.3 | 12 | 0.16 |
| 23 | Zhang, Dai (23) | 97 | 0.8 | 10 | 0.008 | 82 | 1.6 | 10 | 0.02 |
|  | Studies 1-23 |  |  |  | Mean = 0.15 |  |  |  | Mean = 0.26 |

**How these 23 published papers were selected:** A list of highly cited papers was generated using Clarivate Web of Science under the following search term “sucrose preference” AND “rat.” Nine studies were selected based on the following criteria: tested rats (i.e., not mice); avoided sucrose concentrations higher than 1%; and mean, SD or SEM, and n values were stated in the text or could be estimated from a figure. In the same way, 14 additional studies were selected from a meta-analysis on the chronic mild stress procedure by Antoniuk et al. (reference 24 below), doi: <https://doi.org/10.1016/j.neubiorev.2018.12.002>.

**References**

1. Willner P, Towell, A., Sampson, D., Sophokleous, S., & Muscat, R. A. . Reduction of sucrose preference by chronic unpredictable mild stress, and its restoration by a tricyclic antidepressant. Psychopharmacology (Berl). 1987;93(3):358-64.

2. Rygula R, Abumaria N, Flugge G, Fuchs E, Ruther E, Havemann-Reinecke U. Anhedonia and motivational deficits in rats: impact of chronic social stress. Behav Brain Res. 2005;162(1):127-34.

3. Bekris S, Antoniou K, Daskas S, Papadopoulou-Daifoti Z. Behavioural and neurochemical effects induced by chronic mild stress applied to two different rat strains. Behav Brain Res. 2005;161(1):45-59.

4. Forbes NF, Stewart, C. A., Matthews, K., & Reid, I. C. Chronic mild stress and sucrose consumption: validity as a model of depression. Physiology & behavior. 1996;60(6):1481-4.

5. You Z, Luo C, Zhang W, Chen Y, He J, Zhao Q, et al. Pro- and anti-inflammatory cytokines expression in rat's brain and spleen exposed to chronic mild stress: involvement in depression. Behav Brain Res. 2011;225(1):135-41.

6. Sun JD, Liu Y, Yuan YH, Li J, Chen NH. Gap junction dysfunction in the prefrontal cortex induces depressive-like behaviors in rats. Neuropsychopharmacology. 2012;37(5):1305-20.

7. Luo DD, An SC, Zhang X. Involvement of hippocampal serotonin and neuropeptide Y in depression induced by chronic unpredicted mild stress. Brain Res Bull. 2008;77(1):8-12.

8. Hamani C, Machado DC, Hipolide DC, Dubiela FP, Suchecki D, Macedo CE, et al. Deep brain stimulation reverses anhedonic-like behavior in a chronic model of depression: role of serotonin and brain derived neurotrophic factor. Biol Psychiatry. 2012;71(1):30-5.

9. Lu Y, Ho CS, Liu X, Chua AN, Wang W, McIntyre RS, et al. Chronic administration of fluoxetine and pro-inflammatory cytokine change in a rat model of depression. PLoS One. 2017;12(10):e0186700.

10. Li XL, Yuan YG, Xu H, Wu D, Gong WG, Geng LY, et al. Changed Synaptic Plasticity in Neural Circuits of Depressive-Like and Escitalopram-Treated Rats. Int J Neuropsychopharmacol. 2015;18(10):pyv046.

11. Chen J, Wang ZZ, Zuo W, Zhang S, Chu SF, Chen NH. Effects of chronic mild stress on behavioral and neurobiological parameters - Role of glucocorticoid. Horm Behav. 2016;78:150-9.

12. Gill M, Kinra M, Rai A, Chamallamudi MR, Kumar N. Evaluation of antidepressant activity of methanolic extract of Saraca asoca bark in a chronic unpredictable mild stress model. Neuroreport. 2018;29(2):134-40.

13. Song J, Hou, X., Hu, X., Lu, C., Liu, C., Wang, J., Liu, W., Teng, L., & Wang, D. Not only serotonergic system, but also dopaminergic system involved in albiflorin against chronic unpredictable mild stress-induced depression-like behavior in rats. Chemico-biological interactions. 2015;242:211-7.

14. Du H, Wang K, Su L, Zhao H, Gao S, Lin Q, et al. Metabonomic identification of the effects of the Zhimu-Baihe saponins on a chronic unpredictable mild stress-induced rat model of depression. J Pharm Biomed Anal. 2016;128:469-79.

15. Du H, Zhao H, Lai X, Lin Q, Zhu Z, Chai Y, et al. Metabolic profiles revealed synergistically antidepressant effects of lilies and Rhizoma Anemarrhenae in a rat model of depression. Biomed Chromatogr. 2017;31(7).

16. Hui JJ, Xi GJ, Liu SS, Li XL, Geng LY, Teng GJ, et al. Blood oxygen level-dependent signals via fMRI in the mood-regulating circuit using two animal models of depression are reversed by chronic escitalopram treatment. Behav Brain Res. 2016;311:210-8.

17. Qin T, Fang F, Song M, Li R, Ma Z, Ma S. Umbelliferone reverses depression-like behavior in chronic unpredictable mild stress-induced rats by attenuating neuronal apoptosis via regulating ROCK/Akt pathway. Behav Brain Res. 2017;317:147-56.

18. Su GY, Yang JY, Wang F, Ma J, Zhang K, Dong YX, et al. Antidepressant-like effects of Xiaochaihutang in a rat model of chronic unpredictable mild stress. J Ethnopharmacol. 2014;152(1):217-26.

19. Wu GF, Ren S, Tang RY, Xu C, Zhou JQ, Lin SM, et al. Antidepressant effect of taurine in chronic unpredictable mild stress-induced depressive rats. Sci Rep. 2017;7(1):4989.

20. Xi G, Hui J, Zhang Z, Liu S, Zhang X, Teng G, et al. Learning and memory alterations are associated with hippocampal N-acetylaspartate in a rat model of depression as measured by 1H-MRS. PLoS One. 2011;6(12):e28686.

21. Xiong Z, Yang J, Huang Y, Zhang K, Bo Y, Lu X, et al. Serum metabonomics study of anti-depressive effect of Xiao-Chai-Hu-Tang on rat model of chronic unpredictable mild stress. J Chromatogr B Analyt Technol Biomed Life Sci. 2016;1029-1030:28-35.

22. Yang B, Liu Z, Wang Q, Chai Y, Xia P. Pharmacokinetic comparison of seven major bioactive components in normal and depression model rats after oral administration of Baihe Zhimu decoction by liquid chromatography-tandem mass spectrometry. J Pharm Biomed Anal. 2018;148:119-27.

23. Zhang M, Dai W, Liang J, Chen X, Hu Y, Chu B, et al. Effects of UCMS-induced depression on nociceptive behaviors induced by electrical stimulation of the dura mater. Neurosci Lett. 2013;551:1-6.

24. Antoniuk S, Bijata M, Ponimaskin E, Wlodarczyk J. Chronic unpredictable mild stress for modeling depression in rodents: Meta-analysis of model reliability. Neurosci Biobehav Rev. 2019;99:101-16.
