# Supplementary figures and images for: Stable long-term individual differences in 50-kHz vocalization rate and call subtype prevalence in adult male rats: Comparisons with sucrose preference
Source: PLoS One. 2022 Oct 27;17(10):e0276743. doi: 10.1371/journal.pone.0276743 (PMC9612506; doi:10.1371/journal.pone.0276743)

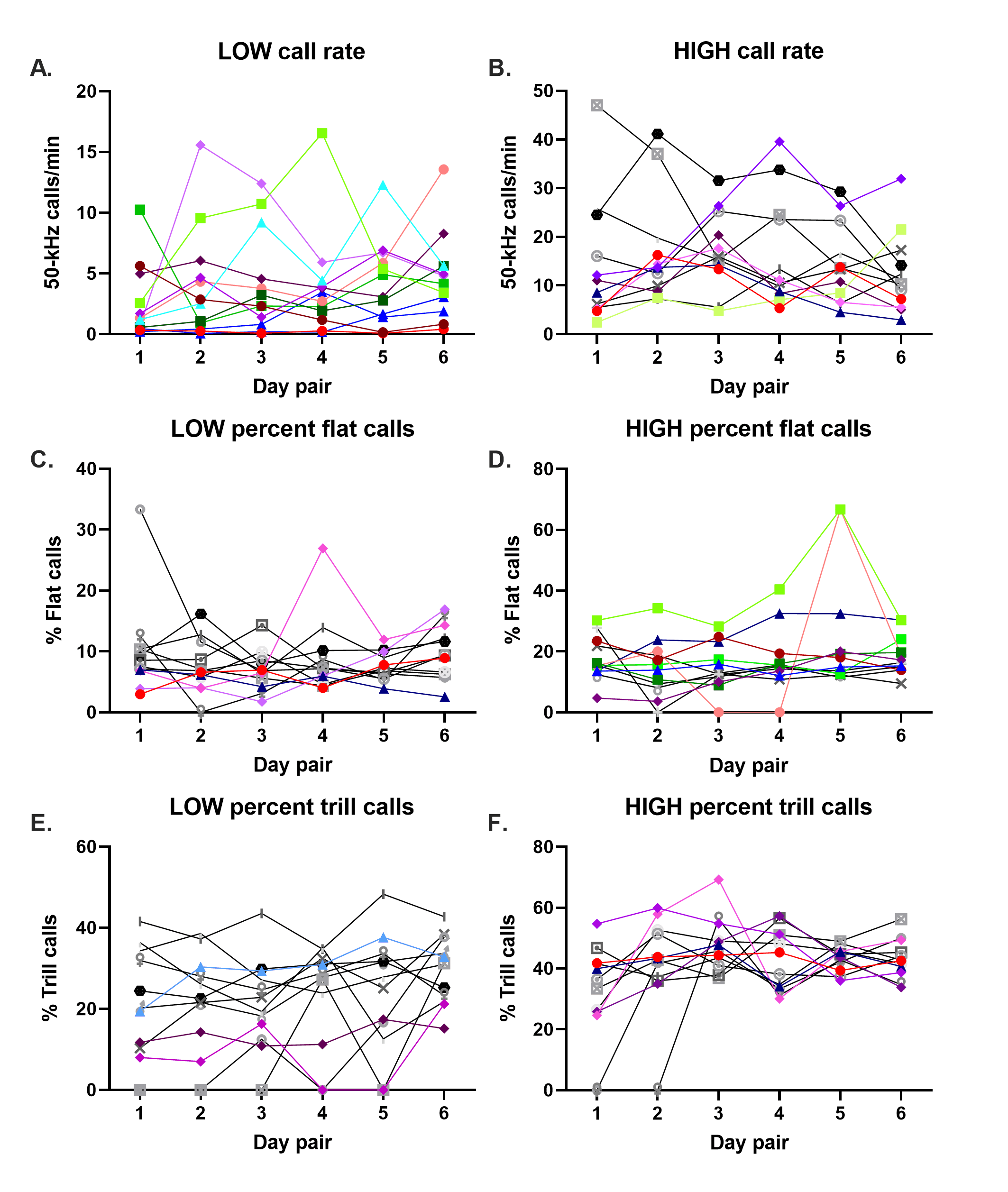

Supplement: S1 Fig — The 24 rats were median-split into low and high subgroups (n = 12), based on the following measures: (A and B) 50-kHz call rate, (C and D) percentage of flat calls, and (E and F) percentage of trill calls. Each line represents an individual rat and each symbol represents a day pair (6 day pairs). (TIF) [file pone.0276743.s004.tif]

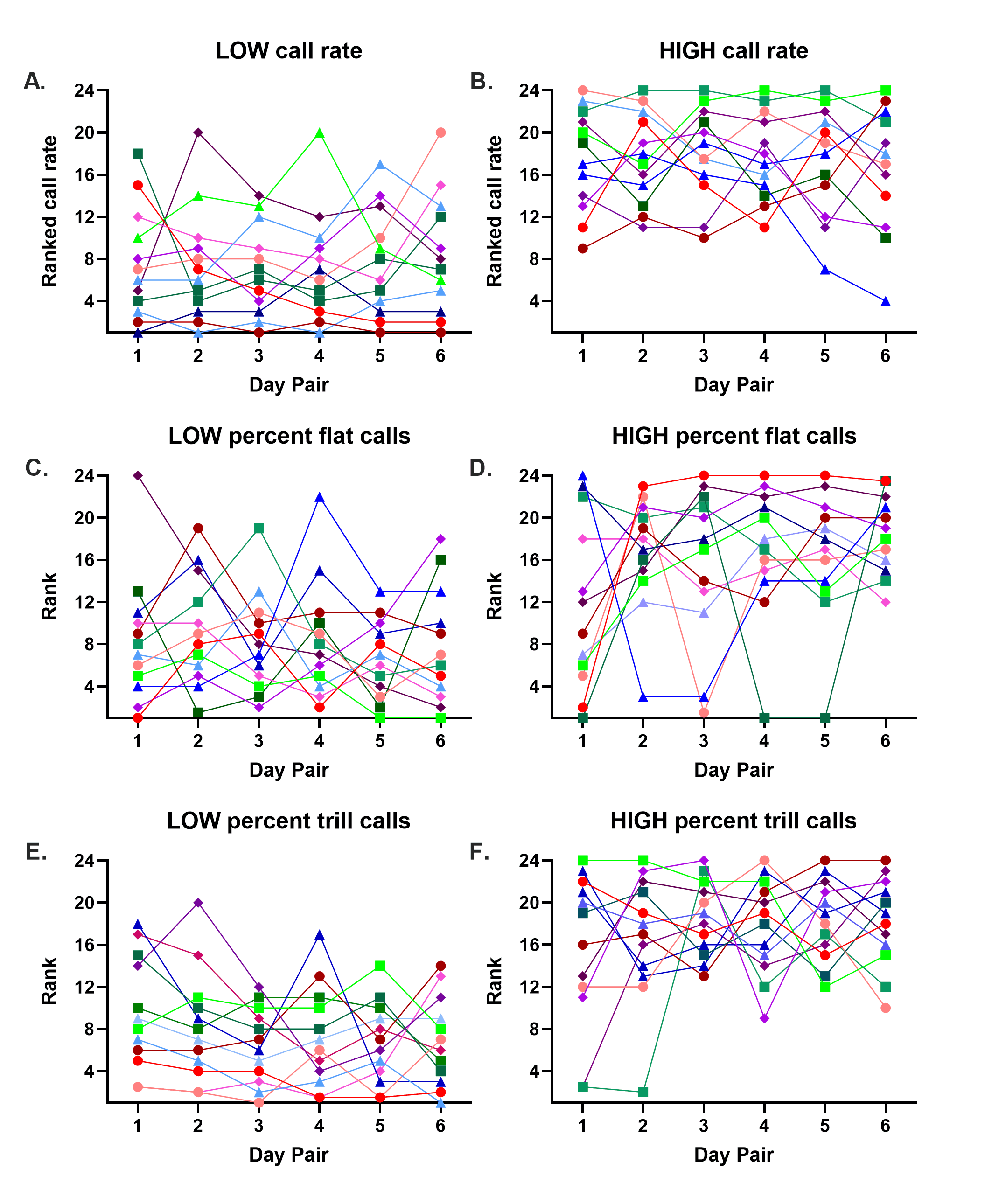

Supplement: S2 Fig — Rats were split into high and low performers based on a median split of each of the three main USV measures. Rats were then ranked on their performance on each measure, for each day pair (1 = lowest performer, 24 = highest performer). (A and B) The ranked 50-kHz calling rate is shown for each rat across the 6 day pairs. Ranked percentage prevalence of (C and D) flat calls and (E and F) trill calls were very stable across the 6 day pairs. Each symbol represents a day pair and each line represents a rat. (TIF) [file pone.0276743.s005.tif]

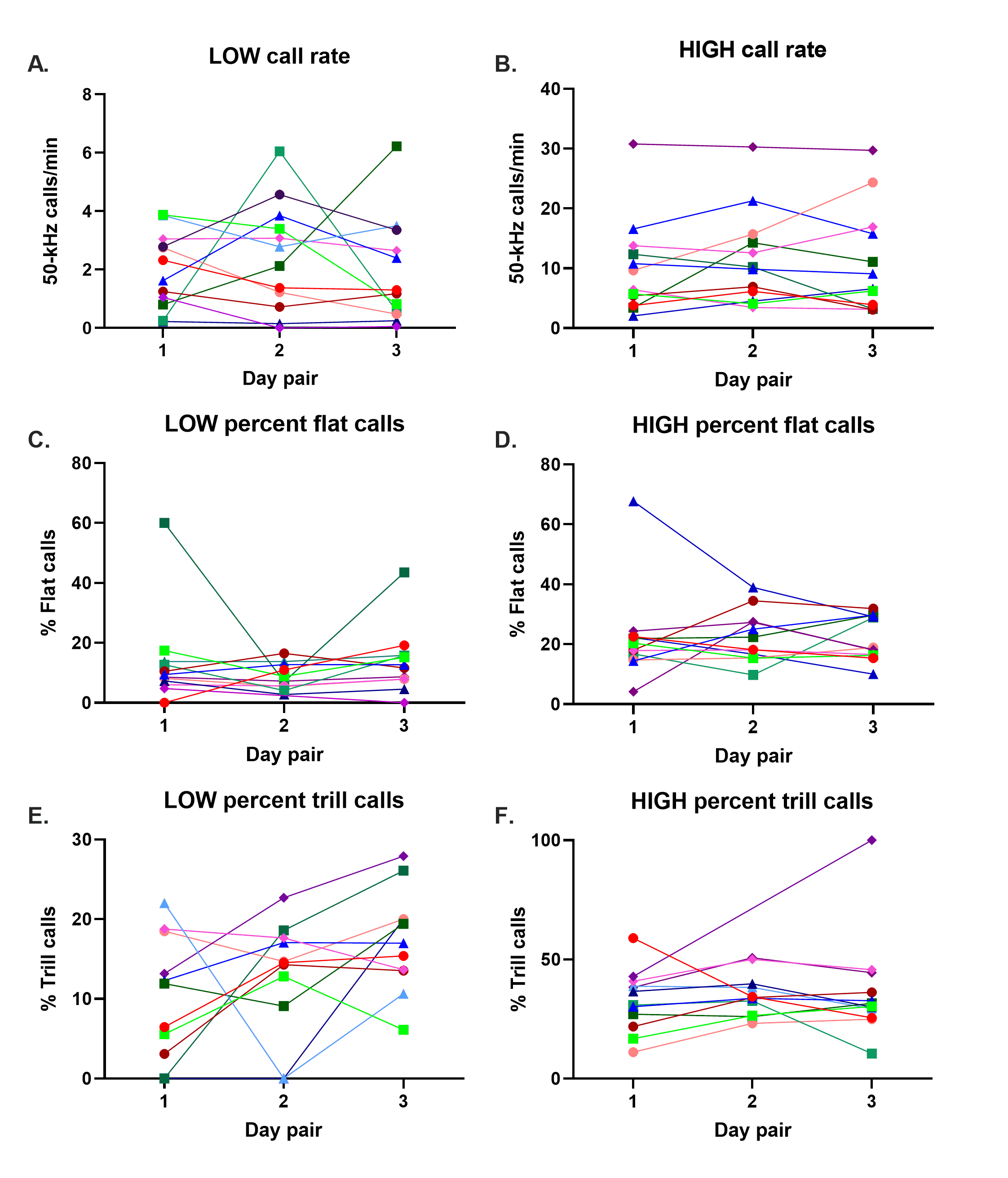

Supplement: S3 Fig — A median split of each USV measure was performed to separate rats into high and low performers. (A and B) The rate of 50-kHz calling is shown for each rat across the 3 day pairs. Percentage of (C and D) flat calls, (E and F) and trill calls (E and F) across the 3 day pairs. Each symbol represents a day pair and each line represents a rat. (TIF) [file pone.0276743.s006.tif]

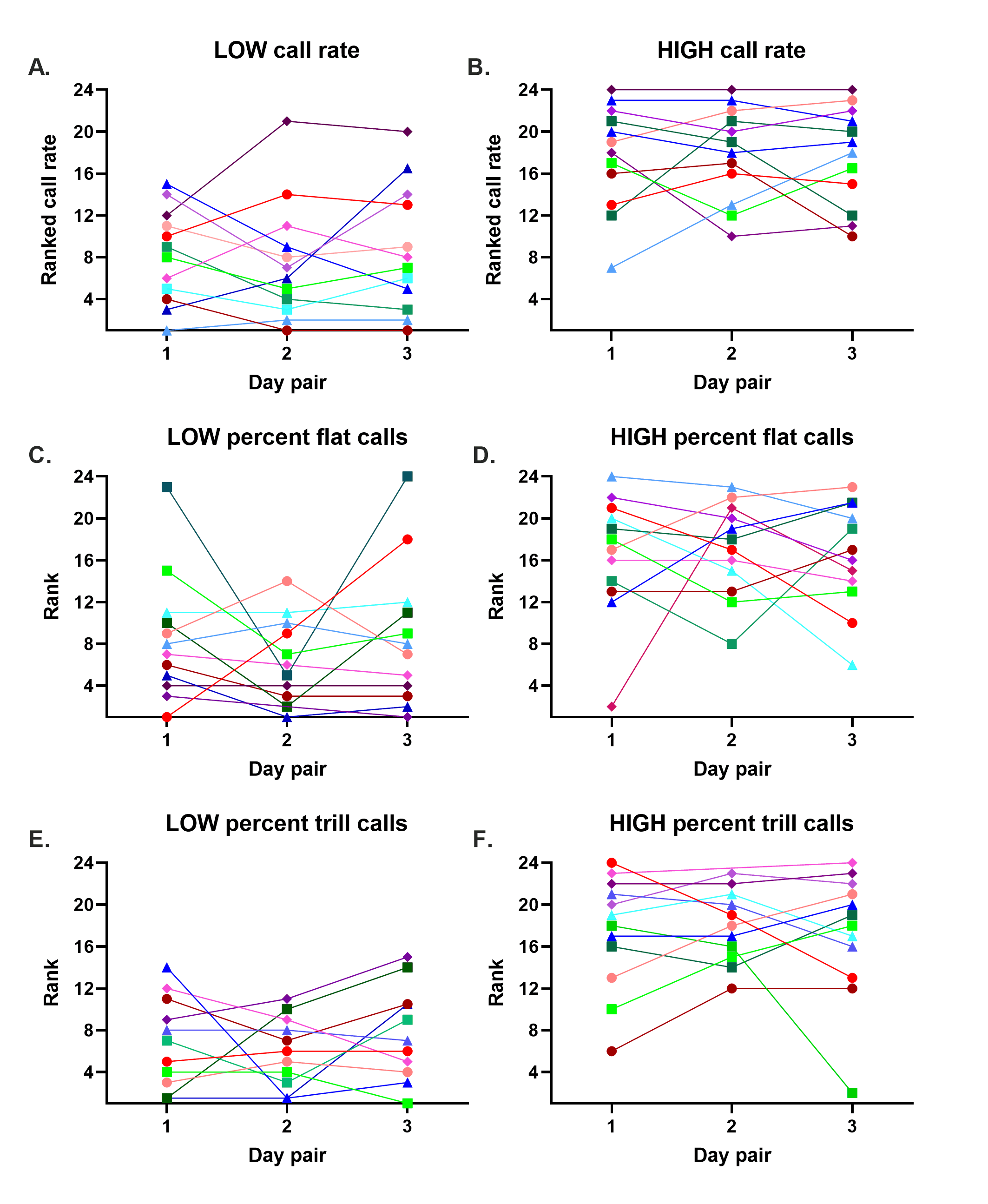

Supplement: S4 Fig — A median split of each USV measure was performed to separate rats into high and low performers. Rats were then ranked on their performance on each measure, for each day pair (1 = lowest performer, 24 = highest performer). (A and B) The ranked 50-kHz calling rate is shown for each rat across the 3 day pairs. Ranked percentage prevalence of (C and D) flat calls, and (E and F) trill calls were very stable across the 3 day pairs. Each symbol represents a day pair and each line represents a rat. (TIF) [file pone.0276743.s007.tif]

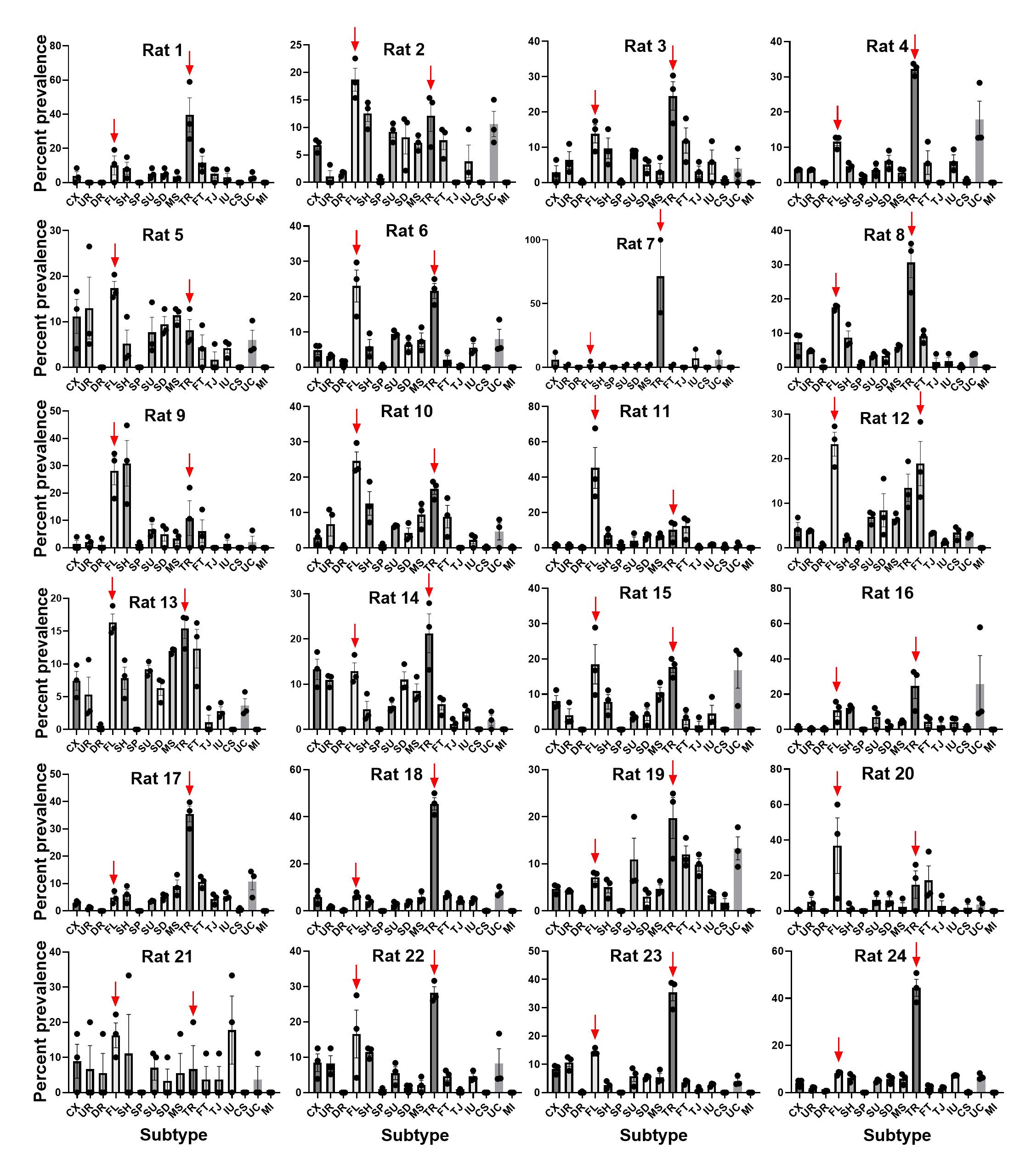

Supplement: S5 Fig — The mean + SEM percentage prevalence of each call subtype is shown (n = 3 day pairs). Most rats show a preference for trill calls over flat calls. Red arrows point to the prevalence of flat and trill calls. Call subtype definitions are as follows; CX: complex, UR: upward ramp, DR: downward ramp, FL: flat, SH: short, SP: split, SU: step-up, SD: step-down, MS: multi-step, TR: trill, FT: flat-trill, TJ: trill with jumps, IU: inverted-U, CS: composite, UC: unclear, MI: miscellaneous. (TIF) [file pone.0276743.s008.tif]

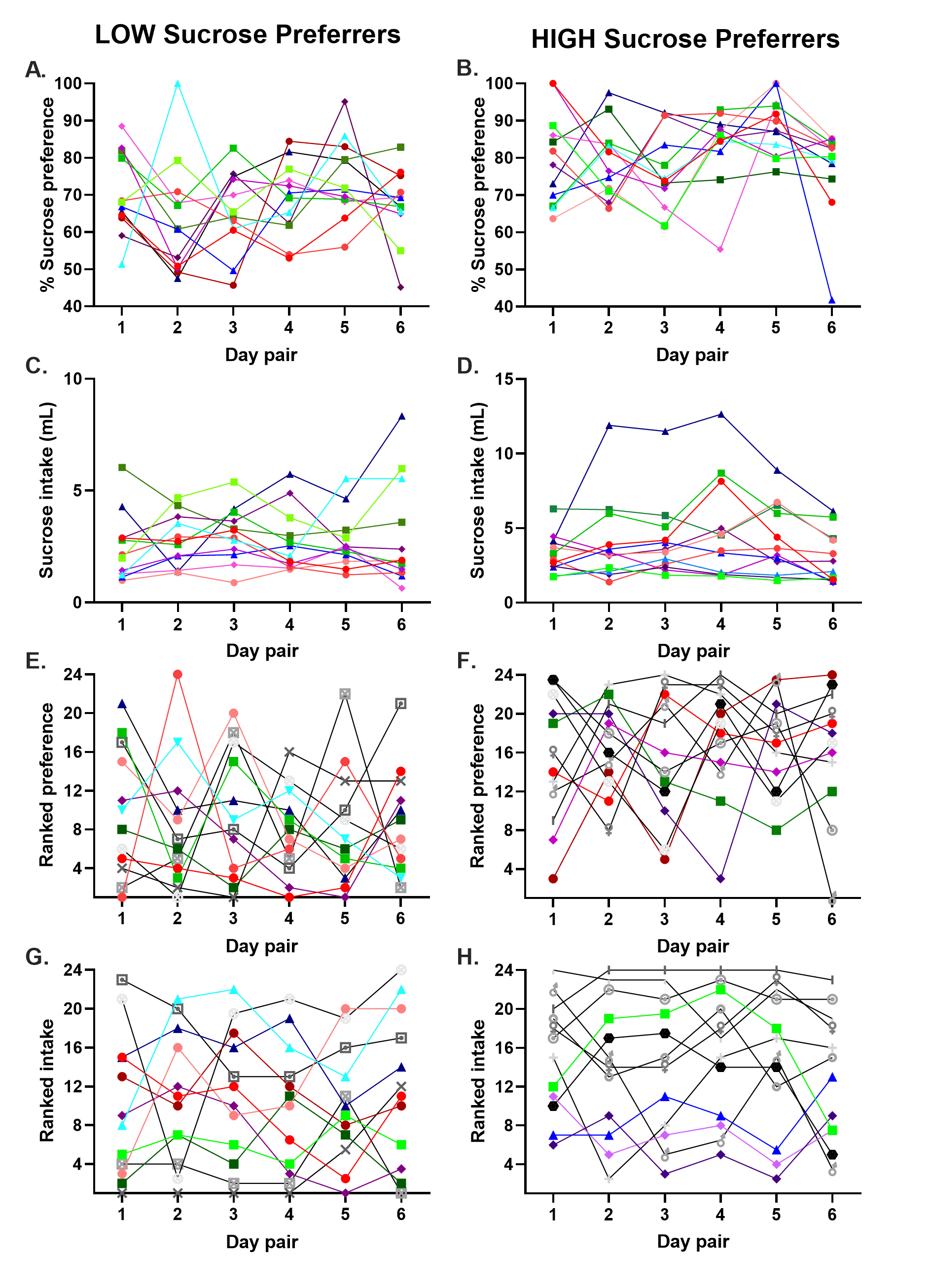

Supplement: S6 Fig — Each rat was tested in 12 test sessions and data were averaged to form 6 day pairs (each symbol represents a day pair and each line represents a rat). The 24 rats were median-split into low and high subgroups (n = 12), based on their percent sucrose preference. Panels A and B show sucrose preference, whereas panels C and D show sucrose intake. Panels E-H show the same data as in panels A-D, except that y-axis values show the ranked score for each rat on a given day pair, for sucrose preference (panels E and F) or sucrose intake (panels G and H). For each day pair, subjects were assigned a rank based on their sucrose preference and sucrose intake (1 = lowest performer, 24 = highest performer). (TIF) [file pone.0276743.s009.tif]

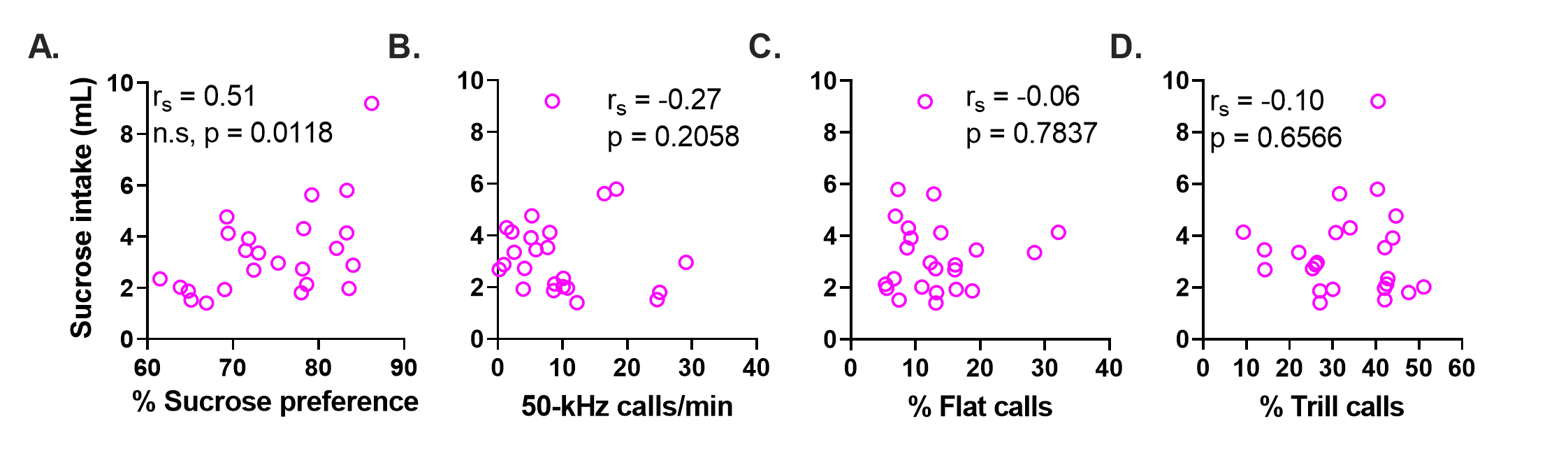

Supplement: S7 Fig — (A) Non-significant trend relating sucrose preference to sucrose intake. (B-D) the lack of relationship between sucrose intake and the three main USV measures, i.e., (B) call rate, percentage of (C) flat calls, (D) percentage of trill calls. Each symbol represents a single rat (n = 24 rats). Spearman correlation coefficients are shown within panels (rs). (TIF) [file pone.0276743.s010.tif]

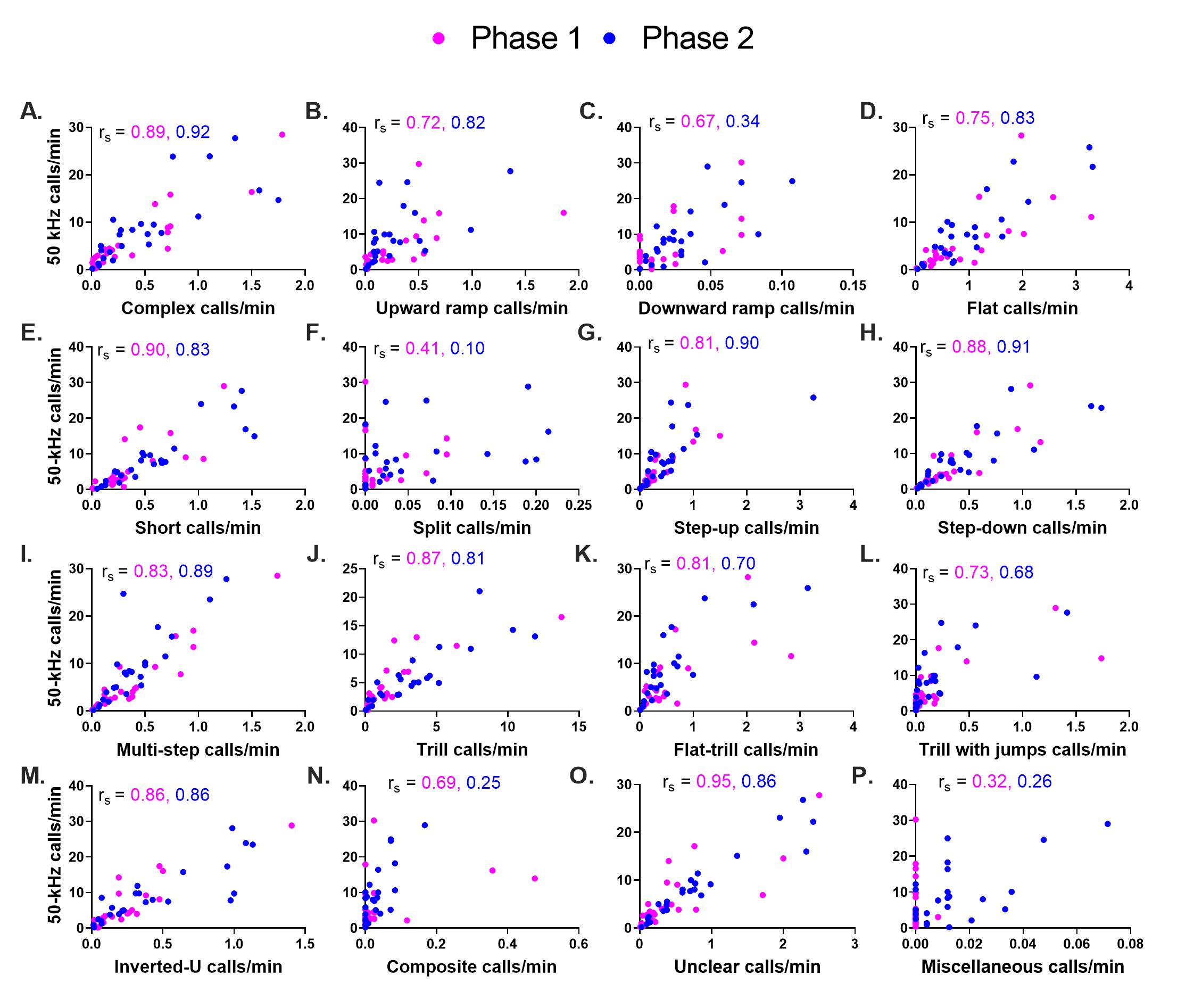

Supplement: S8 Fig — (S8A-P) 50-kHz call rate was calculated exclusively for each subtype by excluding that subtype from the analysis (i.e., using the remaining subtypes). A positive correlation can be observed between 50-kHz call rate and almost all 50-kHz call subtypes. Each symbol represents a single rat (n = 24 rats). Spearman correlation coefficients are shown within panels (rs). (TIF) [file pone.0276743.s011.tif]
