# Supplementary material for: Stable long-term individual differences in 50-kHz vocalization rate and call subtype prevalence in adult male rats: Comparisons with sucrose preference
Source: PLoS One. 2022 Oct 27;17(10):e0276743. doi: 10.1371/journal.pone.0276743 (PMC9612506; doi:10.1371/journal.pone.0276743)
Supplement: Attachment — Submitted filename: PloSone Review.docx [file pone.0276743.s012.docx]

PONE-D-22-12721

The objectives of the indicated study are to observe, in rats, the relationship between Ultrasonic Vocalization (USV) content and sucrose preference (SP) over time under basal conditions (no intervention). Both USV and SP are evidenced to provide information relevant to affect; however, it is uncertain if they are of unique affective dimensions. Further consideration is given to the individual stability of USV and sucrose preference, with further investigation into USV sub-type components. The paper is well-researched and flows well. The statistical analysis is well described, however, there are a couple elements that require further clarification.

Minor Comments

Line 17: Is it sucrose preference or sucrose preference test, be consistent throughout text and figures.

Line 31-33: A greater distinction needs to be made here. The initial statement regarding measures of interest include rate of 50 kHz calls, and relative prevalence of trill and flat call subtypes.

Line 43: Reflected.

Line 46: You need to provide a bit of background on the classification schematic.

Line 47-48: Respectively?

Line 81-82: Reword: “has been little investigated”

Line 106: Were the animals handled prior to experimentation? If so, how much and how long? Were the animals allowed acclimation to the facility prior to the beginning of the experiment?

Line 108-109: How old? Days? This is important for studies concerning USV emission and replicability.

Line 124: Either take out 30 sessions or state 30 sessions: 12 SP, 18 USV.

Line 138: Was there any reason you decided on 13 weeks?

Line 145-146: Any bedding in these cages?

Line 178: Did you assess whether USV in one open field could be detected in another? Where did the testing occur? SP took place in the colony room, did USV as well?

Line 179: Why change half? Are you attempting to provoke USV by scented bedding?

Line 188: Please state the frequency cutoff limit for 50 vs 22 kHz calls. What was the minimum call intensity?

Line 191-192: Explicitly state the length of the trial somewhere above. How long were the habituation trials for USV?

Line 212-213: For the SP, data was removed because it affected results, but it was not removed for the USV because it didn't affect the results. Decide whether outliers will be removed, and on what basis, and then run the analysis. The decision should be made and followed through with before any analysis is completed.

Line 245: Add a comma or "as well as"

Line 302: Respectively?

Line 305: I would suggest taking out the subheadings of phase 1 and phase 2 as most of this is a comparison between the two phases. I think it would also help the flow of the paper to discuss/explain the figures in order highlighting the similarities and differences between the two phases.

Line 356: Write A, B, C after what each describes.

Line 405: Is the difference between absolute and relative that absolute is the number of calls and relative is the value as a percent of total calls? It would be helpful to define this in the methods and clarify.

Lines 475-477: An explanation of how relative prevalence was calculated would be helpful in understanding how it is not skewed by high calling rats (this would be beneficial in the methods).

Major Comments

There are minimal major comments associated with this document.
