# Supplementary material for: Stable long-term individual differences in 50-kHz vocalization rate and call subtype prevalence in adult male rats: Comparisons with sucrose preference
Source: PLoS One. 2022 Oct 27;17(10):e0276743. doi: 10.1371/journal.pone.0276743 (PMC9612506; doi:10.1371/journal.pone.0276743)
Supplement: Attachment — Submitted filename: Adithi_Response to reviewers round 2 FINAL.docx [file pone.0276743.s014.docx]

We would again very much like to thank the reviewer for his comments on the manuscript. As before, our responses are indicated by "AUTH" in the text below (line numbers refer to the revised manuscript). We are in agreement with all comments and believe we have now addressed them satisfactorily.

In addition to the above, we have made a few small edits to improve clarity and we have modified some figures. In terms of figures, we previously displayed only our 14 defined 50-kHz call subtypes, whereas all the call prevalences was calculated using 16 call categories (i.e. the 14 defined subtypes plus "unclear" and "miscellaneous" categories). We have therefore added these two last call categories to the relevant figures (i.e. Fig 7, Fig 10, S5 Fig and S8 Fig). In addition, in order to better reflect our new analysis of inter-call category correlations, we have shifted data previously shown in Fig 10 (panels O and P) to become new panels in Fig 5.

Review Comments to the Author

Please use the space provided to explain your answers to the questions above. You may also include additional comments for the author, including concerns about dual publication, research ethics, or publication ethics. (Please upload your review as an attachment if it exceeds 20,000 characters)

Reviewer #1: (No Response)

**Reviewer #2: The authors have addressed most of my earlier concerns and the paper is much improved. The one major issue I have is with the statistics. In some cases, the authors are correlating the proportion of one type of call with the proportion of another (i.e., percentage of trills against the percentage of flat calls) (e.g., Fig 5 E & F, Fig. 9, Fig 10 O and P). As I explained in my earlier comments, this will tend to over-estimate the relationship between calls in a negative direction. To reiterate, if you have just two categories of calls then the correlation between the proportion of calls will, trivially, be perfectly anticorrelated. If you have three categories of calls, then higher proportions of one call do not guarantee a lower proportion of either of the other two call types, but, on average, there will still be a strong tendency to find a negative correlation. In other words, the correlation of proportions is a *biased estimator*. Hence, we cannot know for certain whether the -.75 correlation between flat and trill calls is due to mathematical necessity or a true tendency of rats that emit lots of trills to also reduce the number of flats. The authors have argued in essence (lines 532-538) that, because not all of their correlations are negative, we can trust the correlations that are. However, with three or more proportions adding to 1, the mathematics doesn’t guarantee that all correlations will be negative. It merely dictates that, on average, one is more likely to find negative correlations. Even if the data is completely random and therefore meaningless, one would still expect to find far more significant negative correlations than one should expect using an unbiased statistic. The use of a biased estimator is invalid and is not appropriate in a published, peer-reviewed paper. My suggestion is to perform these sub-type correlations on the absolute call numbers. If that wipes out all significant effects, then the author can just exclude these results from the manuscript. In my opinion, there are still enough solid results to justify publication.**

**AUTH:** Thank you for pushing us to think harder about this issue of correlations between different 50-kHz call subtypes. As a result, we have expanded our analysis, using two separate approaches in order to establish whether our observed Spearman rho values can readily be explained by the mathematical tendency for percentage measures to be anti-correlated (please see below).

These new analyses are presented in Results (Comparisons between all 50-kHz call subtypes, lines 401- 456), and covered in the Discussion (Methodological considerations, lines 513- 548; Inter-call subtype correlations, lines 617-642).

Most importantly, these analyses have established that the negative correlation that we had reported between the percentage prevalence of flat vs. trill calls remains statistically significant even after controlling for the mathematical anti-correlation issue.

The first of these two analytical approaches was based on collapsing our data into three call categories (i.e. flat, trill, and non-flat/non-trill), and then running simulations of 24-rat experiments based on drawing random numbers from populations having the same group mean and SD percentage prevalence values as our observed call subtype data. In our second approach, we asked whether a high prevalence of flat or trill calls was associated with a disproportionate shift in the prevalence of any particular non-flat/non-trill call subtypes. To this end, we would have liked to apply the same simulation-based approach to our data set comprising the full range of 50-kHz call categories. However, this proved impossible as the less prevalent call subtypes were not normally distributed. Therefore, in our second approach, we instead determined Spearman correlation (rs) coefficients relating flat or trill call prevalence (expressed, as before, as a percentage of *all* 50-kHz calls) to the prevalence of every other 50-kHz call subtype (now expressed as a percentage of *all non-flat/non-trill* 50-kHz calls).

Please note that this second approach, like the first approach, is non-biased, i.e. it avoids the mathematical anti-correlation issue that you raised. This second approach identified statistically significant correlations between certain call subtypes and either flat or trill calls (the results are shown in Table 1 under "Approach 2").

As per your suggestion, we also use the *absolute* call prevalence data in order to calculate correlation coefficients between pairs of 50-kHz call subtypes. This analysis produced positive Spearman rho values (mean rho ± SD = + 0.63 ± 0.18 in Phase 1, for example). However, we consider this type of correlation of only limited value, as such high correlations could easily be driven by differences in the overall 50-kHz call rate. This can be seen as follows, using an extreme example. Suppose that our 24 rats differed in their 50-kHz call rate, but that each rat made exactly 40% trill and 30% flat calls. Now imagine a scatterplot showing the trill call rate vs. the flat call rate, with each rat represented by single dot. In such a scenario, there would be a perfect positive correlation between the absolute prevalence (e.g. calls/min) of flat calls and trill calls.

**A similar argument can be made about the correlation of absolute call numbers with total number of calls (e.g., Table 1 and Fig S8). It should not be a surprise that A is correlated with A+B+C+D. Notice how many of the p values in the “Absolute prevalence” column are significant? Again, this is a biased estimator and is invalid. The authors should just remove these estimates. The other part of Table 1 that looks at the percental prevalence to call rate is valid and can be retained.**

**AUTH**: Apologies for overlooking this point. To avoid this bias, we have now correlated the absolute prevalence (calls/min) of each subtype with the 50-kHz call rate generated from summing all the *remaining* subtypes (e.g., correlating A with B+C+D+E etc). In this new analysis, the *absolute* prevalences of almost all subtypes are still positively correlated with the call rate. While conducting this analysis, we also found that we had not previously accounted for the fact that some rats were time-sampled. We have now corrected this oversight (which pertains only to this analysis), and as a result the correlation coefficients are actually somewhat higher than before, strengthening our conclusion. Please refer to lines 480-486 of the Results and lines 595-605 of the Discussion.

**These statistical concerns are critical and should be addressed before publication. Besides this, I have just a few minor concerns, as listed below.**

**LESSER CONCERNS (Reviewer 2)**

**Fig 4 (and elsewhere): Referents of call type abbreviations (CX, UR, etc) need to be provided**

**AUTH:** Referents have been added for Fig 4, (lines 301-305) and Fig 7 (lines 362- 365).

**Fig 7: The results say that one rat emitted more inverted U calls than any other call type. I cannot see this because the inverted U type is apparently missing from the graph (I assume its abbreviation is IU). I see the justification for leaving out certain low prevalence calls, but, in order to illustrate what the authors are claiming, the IU bar should be retained.**

**AUTH:** We did not identify any of the subtypes as missing from the figures, including IU/inverted-U (Fig 7 and S5 Fig). However, we have clarified this result and referred to the relevant panels in the manuscript (lines 356-358). Overall, two rats preferentially emitted a subtype other than flat or trill calls; short calls in one case (Rat 13 in Fig 7 and Rat 9 in S5 Fig) and inverted-U calls in the other (Rat 21, phase 2, shown in S5 Fig).

**Line 574. As I said in my previous comments, one possible explanation for the lack of correlation between SP and any of your vocalization measures could be that your range of SP values is too limited. If all your SP values were identical, then obviously, you’d never find a correlation with any measure. Similarly, when your range of values is small, your ability to detect correlations with other measures is reduced. Other studies, which include an independent variable (i.e., drug vs control) might have a larger range of SP values and hence be better able to detect a correlation between SP and vocalizations. The authors have added the statement “there was substantial inter-rat variability in both SP and USV measures, advantageous for correlational analysis.” I don’t feel that this goes far enough, as this issue goes to the heart of the paper. I suggest that the authors compare the coefficient of variation in their study with that in other studies of SP (to the best of their ability). Cite some ranges from other studies for comparison. If the range in your data is comparable to other studies that include an experimental manipulation, then you can largely put this concern to rest. If other studies have a much larger range, however, then the authors need to include a major caveat in interpreting their null result. I do see that the authors offer two other reasons to trust their null result: the low Spearman’s rho values and the near-significant correlation with sucrose consumption. If the range in the data is low, this would also cause low Spearman’s rho values. Further, given that sucrose preference and total sucrose consumption are measuring different aspects of the same task, I would expect these values to be strongly related, so their near-significant correlation doesn’t convince me that power is sufficient to find weaker effects.**

**AUTH:** In order to compare the CV values from our study to the literature, we initially used Clarivate Web of Science to search for the most cited papers under the following search term “sucrose preference” AND “rat.” Nine studies were selected based on the following criteria: tested rats (i.e., not mice); avoided sucrose concentrations higher than 1%; and mean, SD or SEM, and n values were stated in the text or could be estimated from a figure. In the same way, 14 additional studies were selected from a meta-analysis on the chronic mild stress procedure by Antoniuk et al. (2019), doi: <https://doi.org/10.1016/j.neubiorev.2018.12.002>.

In our own study, we report a coefficient of variation (CV) of 0.10 for our sucrose preference data (line 381). Published studies generally yielded higher CV values (control group mean CV = 0.15 and stressed group mean CV = 0.26; see new S3 Table and reference list below). However, most of these published studies reported sucrose preference data from a single session, or at best a few test sessions, such that it is unclear how much of the observed variability represented random noise vs. consistent individual differences. In our study, in contrast, the sucrose preference test was conducted numerous times on each animal, so that our sucrose preference values are likely to be more reliable, and our CV value less reflective of random noise. These data have been included as a supplementary table (S3 Table) and covered in the Discussion section of the manuscript (lines 553-560, and lines 664-668).

**Line 487: “rats were tested in the home-cage environment for 4 days”. I questioned this statement in my previous comments and the authors have responded “We feel our statement is justified.” I am not looking for justification. I simply don’t know what data you are referring to. Looking at Figure 1, I see habituation days and testing days, but no reference to home-cage testing. Similarly, in the methods, there is no mention of home-cage testing. Were there unreported recordings done in the home cage, or does “home-cage” refer to some of the testing days in Figure 1. This need to be clarified.**

**AUTH:** We agree that this wording was confusing. We did not test the rats in a home-cage environment at any point in our study. As we now make clear, we were actually referring to the Schwarting et al. 2007 study mentioned in the immediately preceding sentence (lines 569-570). More specifically, Schwarting et al. 2007 recorded a group of rats in their home cages (albeit in a separate testing room). The data for home cage recorded USV emission can be seen in Figures 2-4 in their paper (<https://doi.org/10.1016/j.bbr.2007.01.029>). This was the only paper we found that had measured emission of USVs without an eliciting stimulus (ex: drugs or play), and was therefore comparable to our study which also measured USVs in a non-evoked state.

**References**

Antoniuk, S., Bijata, M., Ponimaskin, E., & Wlodarczyk, J. (2019). Chronic unpredictable mild stress for modeling depression in rodents: Meta-analysis of model reliability. *Neurosci Biobehav Rev*, *99*, 101-116. <https://doi.org/10.1016/j.neubiorev.2018.12.002>

Bekris, S., Antoniou, K., Daskas, S., & Papadopoulou-Daifoti, Z. (2005). Behavioural and neurochemical effects induced by chronic mild stress applied to two different rat strains. *Behav Brain Res*, *161*(1), 45-59. <https://doi.org/10.1016/j.bbr.2005.01.005>

Chen, J., Wang, Z. Z., Zuo, W., Zhang, S., Chu, S. F., & Chen, N. H. (2016). Effects of chronic mild stress on behavioral and neurobiological parameters - Role of glucocorticoid. *Horm Behav*, *78*, 150-159. <https://doi.org/10.1016/j.yhbeh.2015.11.006>

Du, H., Wang, K., Su, L., Zhao, H., Gao, S., Lin, Q., Ma, X., Zhu, B., Dong, X., & Lou, Z. (2016). Metabonomic identification of the effects of the Zhimu-Baihe saponins on a chronic unpredictable mild stress-induced rat model of depression. *J Pharm Biomed Anal*, *128*, 469-479. <https://doi.org/10.1016/j.jpba.2016.06.019>

Du, H., Zhao, H., Lai, X., Lin, Q., Zhu, Z., Chai, Y., & Lou, Z. (2017). Metabolic profiles revealed synergistically antidepressant effects of lilies and Rhizoma Anemarrhenae in a rat model of depression. *Biomed Chromatogr*, *31*(7). <https://doi.org/10.1002/bmc.3923>

Forbes, N. F., Stewart, C. A., Matthews, K., & Reid, I. C. (1996). Chronic mild stress and sucrose consumption: validity as a model of depression. *Physiology & Behavior*, *60*(6), 1481-1484.

Gill, M., Kinra, M., Rai, A., Chamallamudi, M. R., & Kumar, N. (2018). Evaluation of antidepressant activity of methanolic extract of Saraca asoca bark in a chronic unpredictable mild stress model. *Neuroreport*, *29*(2), 134-140. <https://doi.org/10.1097/WNR.0000000000000944>

Hamani, C., Machado, D. C., Hipolide, D. C., Dubiela, F. P., Suchecki, D., Macedo, C. E., Tescarollo, F., Martins, U., Covolan, L., & Nobrega, J. N. (2012). Deep brain stimulation reverses anhedonic-like behavior in a chronic model of depression: role of serotonin and brain derived neurotrophic factor. *Biol Psychiatry*, *71*(1), 30-35. <https://doi.org/10.1016/j.biopsych.2011.08.025>

Hui, J. J., Xi, G. J., Liu, S. S., Li, X. L., Geng, L. Y., Teng, G. J., Nie, B. B., Shan, B. C., Yan, J., Dong, L., Reynolds, G. P., & Zhang, Z. J. (2016). Blood oxygen level-dependent signals via fMRI in the mood-regulating circuit using two animal models of depression are reversed by chronic escitalopram treatment. *Behav Brain Res*, *311*, 210-218. <https://doi.org/10.1016/j.bbr.2016.05.044>

Li, X. L., Yuan, Y. G., Xu, H., Wu, D., Gong, W. G., Geng, L. Y., Wu, F. F., Tang, H., Xu, L., & Zhang, Z. J. (2015). Changed Synaptic Plasticity in Neural Circuits of Depressive-Like and Escitalopram-Treated Rats. *Int J Neuropsychopharmacol*, *18*(10), pyv046. <https://doi.org/10.1093/ijnp/pyv046>

Lu, Y., Ho, C. S., Liu, X., Chua, A. N., Wang, W., McIntyre, R. S., & Ho, R. C. (2017). Chronic administration of fluoxetine and pro-inflammatory cytokine change in a rat model of depression. *PLoS One*, *12*(10), e0186700. <https://doi.org/10.1371/journal.pone.0186700>

Luo, D. D., An, S. C., & Zhang, X. (2008). Involvement of hippocampal serotonin and neuropeptide Y in depression induced by chronic unpredicted mild stress. *Brain Res Bull*, *77*(1), 8-12. <https://doi.org/10.1016/j.brainresbull.2008.05.010>

Qin, T., Fang, F., Song, M., Li, R., Ma, Z., & Ma, S. (2017). Umbelliferone reverses depression-like behavior in chronic unpredictable mild stress-induced rats by attenuating neuronal apoptosis via regulating ROCK/Akt pathway. *Behav Brain Res*, *317*, 147-156. <https://doi.org/10.1016/j.bbr.2016.09.039>

Rygula, R., Abumaria, N., Flugge, G., Fuchs, E., Ruther, E., & Havemann-Reinecke, U. (2005). Anhedonia and motivational deficits in rats: impact of chronic social stress. *Behav Brain Res*, *162*(1), 127-134. <https://doi.org/10.1016/j.bbr.2005.03.009>

Song, J., Hou, X., Hu, X., Lu, C., Liu, C., Wang, J., Liu, W., Teng, L., & Wang, D. (2015). Not only serotonergic system, but also dopaminergic system involved in albiflorin against chronic unpredictable mild stress-induced depression-like behavior in rats. *Chemico-biological interactions*, *242*, 211-217. <https://doi.org/https://doi.org/10.1016/j.cbi.2015.10.001>

Su, G. Y., Yang, J. Y., Wang, F., Ma, J., Zhang, K., Dong, Y. X., Song, S. J., Lu, X. M., & Wu, C. F. (2014). Antidepressant-like effects of Xiaochaihutang in a rat model of chronic unpredictable mild stress. *J Ethnopharmacol*, *152*(1), 217-226. <https://doi.org/10.1016/j.jep.2014.01.006>

Sun, J. D., Liu, Y., Yuan, Y. H., Li, J., & Chen, N. H. (2012). Gap junction dysfunction in the prefrontal cortex induces depressive-like behaviors in rats. *Neuropsychopharmacology*, *37*(5), 1305-1320. <https://doi.org/10.1038/npp.2011.319>

Willner, P., Towell, A., Sampson, D., Sophokleous, S., & Muscat, R. A. . (1987). Reduction of sucrose preference by chronic unpredictable mild stress, and its restoration by a tricyclic antidepressant. *Psychopharmacology (Berl)*, *93*(3), 358-364.

Wu, G. F., Ren, S., Tang, R. Y., Xu, C., Zhou, J. Q., Lin, S. M., Feng, Y., Yang, Q. H., Hu, J. M., & Yang, J. C. (2017). Antidepressant effect of taurine in chronic unpredictable mild stress-induced depressive rats. *Sci Rep*, *7*(1), 4989. <https://doi.org/10.1038/s41598-017-05051-3>

Xi, G., Hui, J., Zhang, Z., Liu, S., Zhang, X., Teng, G., Chan, K. C., Wu, E. X., Nie, B., Shan, B., Li, L., & Reynolds, G. P. (2011). Learning and memory alterations are associated with hippocampal N-acetylaspartate in a rat model of depression as measured by 1H-MRS. *PLoS One*, *6*(12), e28686. <https://doi.org/10.1371/journal.pone.0028686>

Xiong, Z., Yang, J., Huang, Y., Zhang, K., Bo, Y., Lu, X., Su, G., Ma, J., Yang, J., Zhao, L., & Wu, C. (2016). Serum metabonomics study of anti-depressive effect of Xiao-Chai-Hu-Tang on rat model of chronic unpredictable mild stress. *J Chromatogr B Analyt Technol Biomed Life Sci*, *1029-1030*, 28-35. <https://doi.org/10.1016/j.jchromb.2016.06.044>

Yang, B., Liu, Z., Wang, Q., Chai, Y., & Xia, P. (2018). Pharmacokinetic comparison of seven major bioactive components in normal and depression model rats after oral administration of Baihe Zhimu decoction by liquid chromatography-tandem mass spectrometry. *J Pharm Biomed Anal*, *148*, 119-127. <https://doi.org/10.1016/j.jpba.2017.09.031>

You, Z., Luo, C., Zhang, W., Chen, Y., He, J., Zhao, Q., Zuo, R., & Wu, Y. (2011). Pro- and anti-inflammatory cytokines expression in rat's brain and spleen exposed to chronic mild stress: involvement in depression. *Behav Brain Res*, *225*(1), 135-141. <https://doi.org/10.1016/j.bbr.2011.07.006>

Zhang, M., Dai, W., Liang, J., Chen, X., Hu, Y., Chu, B., Pan, M., Dong, Z., & Yu, S. (2013). Effects of UCMS-induced depression on nociceptive behaviors induced by electrical stimulation of the dura mater. *Neurosci Lett*, *551*, 1-6. <https://doi.org/10.1016/j.neulet.2013.04.038>
